# Efficacy and Safety of Vasopressin Alone or in Combination With Catecholamines in the Treatment of Septic Shock: A Systematic Review

**DOI:** 10.7759/cureus.29143

**Published:** 2022-09-14

**Authors:** Naishal Mandal, Nang I Kham, Rabia Shahid, Shaili S Naik, Shivana Ramphall, Swarnima Rijal, Vishakh Prakash, Heba Ekladios, Jiya Mulayamkuzhiyil Saju, Sathish Venugopal

**Affiliations:** 1 Internal Medicine, California Institute of Behavioral Neurosciences & Psychology, Fairfield, USA; 2 Internal Medicine, Surat Municipal Institute of Medical Education & Research (SMIMER) Hospital & Medical College, Surat, IND; 3 Research, American University of Antigua, Osbourn, ATG; 4 Internal Medicine, Government Medical College Kozhikode, Kozhikode, IND; 5 Psychiatry, California Institute of Behavioral Neurosciences & Psychology, Fairfield, USA; 6 Internal Medicine, Sree Narayana Institute of Medical Sciences, Ernakulam, IND; 7 General Surgery, Government Medical College, Trivandrum, Trivandrum, IND

**Keywords:** terlipressin, vasopressin, vasoconstrictors, catecholamine, septic shock

## Abstract

Septic shock is one of the life-threatening emergencies in hospital settings. Patients with septic shock have been treated with various vasopressors alone as a first-line or in combination with other agents to improve blood pressure and increase the chance of survival. Our study focuses particularly on the efficacy and safety of vasopressin (VP) alone and in combination with other vasopressors. Our study used Preferred Reporting Items for Systematic Reviews and Meta-Analyses (PRISMA) guidelines, 2020 to do our systematic review. We searched thoroughly for articles in PubMed, PubMed Central (PMC), Medline, and ScienceDirect. To locate all pertinent papers, we employed the medical subject headings (MeSH) systematic search technique. Twelve papers that were related to the study's issue and passed the quality check were extracted after we applied inclusion/exclusion criteria and reviewed the titles and abstracts. We used a variety of assessment methods for diverse study designs as a quality check. We compared all included studies after reviewing them thoroughly. VP and its synthetic variants (Terlipressin and Selepressin) have always been given as adjuvants to catecholamine, especially with Noradrenaline, in low to moderate doses with continuous infusion in patients with septic shock. Furthermore, VP is a better adjuvant agent than Dopamine and Dobutamine. Though VP has been proven superior to other vasopressors as an adjuvant agent in patients with septic shock, it can cause digital ischemia in high doses.

## Introduction and background

Septic shock is a life-threatening medical emergency and a leading cause of death in the United States. Mortality owing to sepsis is about 50% [1]. Most of the deaths in septic shock take place outside the Intensive Care Unit (ICU). One of the studies shows the incidence of severe sepsis is about 300 cases per 100,000 population [1]. Mortality is high in Immunocompromised patients having septic shock. Therefore, rapid aggressive treatment is imperative in such cases [2,3].

In simple words, sepsis is an exaggerated response of the body to infection. Sepsis is defined as a combination of systemic inflammatory response syndrome (SIRS) criteria and the presence of infection. Four parameters are there, out of which two must be there to define SIRS. Those four parameters are temperature >38°C or <36°C, white blood cell count >12,000/mm³ or <4,000/mm³ or >10% bands, heart rate >90, and respiratory rate >20 or PaCO2 <32 mmHg [4]. The presence or absence of organ failure decides the severity of sepsis. Septic shock is defined as sepsis + hypotension. Risk factors for sepsis include aging, immunocompromised state, organ transplant, bone marrow transplant, corticosteroids, prolonged hospital stay, and many more.

Fluids and vasopressors in addition to antibiotics are the mainstays of treatment in septic shock. To maintain an adequate Mean Arterial Pressure (MAP) in septic shock patients, vasopressor therapy is mandatory [4]. Among vasopressors, Norepinephrine (NE) is used as a primary agent to maintain blood pressure over vasopressin (VP) despite having multiple side effects including cardiovascular toxicity, peripheral ischemia due to marked vasoconstriction, impaired immune response, and coagulation [5,6]. Furthermore, non-responsiveness in many patients requiring large doses leads to an increased chance of such adverse effects [7,8].

Recently, numerous research has been conducted to replace NE with VP [8-10]. There are some studies that preferred VP alone or as an adjunctive to NE over NE alone due to the relative deficiency of VP in septic shock [11]. The following are some claimed advantages of VP over NE: Administration of VP decreases the duration of adjuvant vasopressor therapy in septic shock patients and reduces the chance of acute renal injury [12-15]. Therefore, it decreases the adverse effects of NE and has a beneficial effect in combination with corticosteroids [16]. VP receptors are of three types: V1a, V1b, and V2. The V1a-in vessels and liver, V1b-in anterior pituitary where it releases Corticotropin Releasing Hormone (CRH) which ultimately releases adreno-cortico-tropic hormone (ACTH) and V2-found in the kidney [17-19]. Furthermore, VP has a significant effect on reducing inflammatory cytokines compared with NE [20].

This systematic review emphasizes mainly on efficacy and safety of VP alone or in combination with catecholamine in patients with septic shock. Further studies are warranted to know more about the benefits of VP for clinical use.

## Review

Method

We conducted this systematic review with the Preferred Reporting Items for Systematic Reviews and Meta-Analyses (PRISMA) guidelines, 2020 [21]. Our study focused on identifying the efficacy and safety of VP alone or in combination with catecholamines.

Literature Search Strategy

We have searched the following four databases thoroughly - Pubmed, PubMed Central (PMC), Medline, and ScienceDirect for articles that compared VP and catecholamines in the management of septic shock. We used the Medical Subject Headings (MeSH) systemic search strategy to find all relevant articles. The keywords included “septic shock”, “catecholamines” and “vasoconstrictors”. Using the Boolean method, we used combinations of keywords and the MeSH strategy to retrieve all the pertinent articles from the databases. The detailed search strategy for the databases has been stated in Table 1.

**Table 1:** Detailed literature search strategy

Database	Strategy	Number of results before inclusion/ exclusion criteria	Results after inclusion/ exclusion criteria: (Studies from last 10 years, adult population)
PubMed, PubMed Central (PMC), Medline	1] (( "Catecholamines/administration and dosage"[Majr] OR "Catecholamines/adverse effects"[Majr] OR "Catecholamines/drug effects"[Majr] OR "Catecholamines/physiology"[Majr] OR "Catecholamines/therapeutic use"[Majr] OR "Catecholamines/therapy"[Majr] OR "Catecholamines/toxicity"[Majr] )) AND (( "Shock, Septic/complications"[Majr] OR "Shock, Septic/diagnosis"[Majr] OR "Shock, Septic/drug therapy"[Majr] OR "Shock, Septic/physiopathology"[Majr] OR "Shock, Septic/prevention and control"[Majr] OR "Shock, Septic/therapy"[Majr] ))	438	20
	2] (( "Shock, Septic/complications"[Majr] OR "Shock, Septic/diagnosis"[Majr] OR "Shock, Septic/drug therapy"[Majr] OR "Shock, Septic/physiopathology"[Majr] OR "Shock, Septic/prevention and control"[Majr] OR "Shock, Septic/therapy"[Majr] )) AND (( "Vasoconstrictor Agents/adverse effects"[Majr] OR "Vasoconstrictor Agents/classification"[Majr] OR "Vasoconstrictor Agents/drug therapy"[Majr] OR "Vasoconstrictor Agents/pharmacology"[Majr] OR "Vasoconstrictor Agents/therapeutic use"[Majr] OR "Vasoconstrictor Agents/therapy"[Majr] OR "Vasoconstrictor Agents/toxicity"[Majr] ))	471	38
	3] ((( "Vasoconstrictor Agents/adverse effects"[Majr] OR "Vasoconstrictor Agents/classification"[Majr] OR "Vasoconstrictor Agents/drug therapy"[Majr] OR "Vasoconstrictor Agents/pharmacology"[Majr] OR "Vasoconstrictor Agents/therapeutic use"[Majr] OR "Vasoconstrictor Agents/therapy"[Majr] OR "Vasoconstrictor Agents/toxicity"[Majr] )) AND (( "Catecholamines/administration and dosage"[Majr] OR "Catecholamines/adverse effects"[Majr] OR "Catecholamines/drug effects"[Majr] OR "Catecholamines/physiology"[Majr] OR "Catecholamines/therapeutic use"[Majr] OR "Catecholamines/therapy"[Majr] OR "Catecholamines/toxicity"[Majr] ))) AND (( "Shock, Septic/complications"[Majr] OR "Shock, Septic/diagnosis"[Majr] OR "Shock, Septic/drug therapy"[Majr] OR "Shock, Septic/physiopathology"[Majr] OR "Shock, Septic/prevention and control"[Majr] OR "Shock, Septic/therapy"[Majr] ))	149	7
ScienceDirect	(((septic shock) OR (sepsis) OR (severe sepsis) AND ((vasopressors) OR (vasopressin) OR (norepinephrine) OR (catecholamine)) AND ("adults"))	13509	3142

Inclusion Criteria

We have included papers from the last 15 years, which have been published in English Language. Our papers focus on adult population and are relevant to the title.

Exclusion Criteria

Papers discussing pediatric population (below 19 years), unpublished literature, and grey literature have been excluded from our study. 

Data Extraction

All the articles were screened by title, abstract and full text. We extracted data on an excel sheet. The information extracted from articles included years of publication, use, and adverse effects of VP, NE, Dopamine, and Dobutamine.

Quality Appraisal of Included Studies

We used various tools for various study designs which are as follows:

 > Joanna Briggs Institute (JBI) tool for Case-Control study, Cohort study, Cross-sectional study, Case report, and Case series.

> Amster checklist for Systematic Review and Meta-analysis.

> Cochrane bias assessment tool for Randomized Control Trials (RCTs).

In order to undertake this systematic literature review, the papers that met >70% of the criteria were ultimately examined in-depth, as shown in Table 2.

**Table 2:** A quality appraisal of included studies U/C - Unclear, N/A - Not Applicable, HIGH - High chances of bias, LOW - Low chances of bias

Quality appraisal of included studies											
Case-control study											
	Disease group compared with the control group?	Matching between cases and controls?	Used same criteria for the identification of cases and controls?	Measured exposure in a valid and reliable way?	Measured exposure in the same way for cases and controls?	Confounding factors looked for?	What about strategies to deal with confounding factors?	Measured outcomes in a valid and reliable way?	Did the exposure period of interest long enough?	Used appropriate statistical analysis?	
Nascente et al. [22]	YES	YES	YES	YES	YES	YES	U/C	YES	YES	YES	
Cohort study											
	Groups are similar and from the same population?	Measured exposure Similarly, to assign people in both groups?	Measured exposure in a valid and reliable way?	Confounding factors looked for?	Strategies to deal with confounding factors?	Groups were free of outcome in the beginning?	Measured outcomes in a valid and reliable way?	Follow-up time and their duration sufficient?	Follow-up completed?	Strategy for an incomplete follow-up?	Used appropriate statistical analysis?
Fawzy et al. [23]	YES	YES	YES	YES	U/C	YES	YES	U/C	YES	N/A	YES
Vail et al. [24]	YES	YES	YES	YES	U/C	YES	YES	N/A	N/A	N/A	YES
Cross-sectional study											
	Inclusion criteria clearly defined?	Described study subjects and the setting in detail?	Measured Exposure in a valid and reliable way?	Used objective and standard criteria?	Confounding factors?	Strategies to deal with confounding factors?	Measured Outcomes in a valid and reliable way?	Appropriate statistical analysis?			
Yerke et al. [25]	YES	YES	N/A	YES	YES	N/A	YES	U/C			
Case report											
	The patient’s demographic Characteristic?	Patient’s history described and presented as a timeline?	Current clinical condition of the patient?	Diagnostic tests and the results?	Interventionor treatment procedure?	post-intervention clinical condition?	Adverse events (harms) or unanticipated events identify?	Takeaway lessons?			
Ruffin et al. [26]	U/C	YES	YES	U/C	YES	YES	YES	YES			
Case series											
	Inclusion criteria?	Measured condition in a standard, reliable way?	Valid methods used for identification of the condition?	The consecutive inclusion of participants?	Complete inclusion of participants?	Demographics of participants?	Clinical information of the participants?	Outcomes or follow-up results?	Reporting of the presenting site(s)/clinic(s) demographic information?	Appropriate statistical analysis?	
Kny et al. [27]	YES	YES	YES	U/C	YES	YES	YES	YES	U/C	U/C	
Hallengren et al. [28]	YES	YES	YES	U/C	YES	YES	YES	YES	U/C	U/C	
Systematic review and meta-analysis											
	Prior design?	duplicate study selection and data extraction?	Detail literature search?	Grey literature used?	Inclusion and Exclusion criteria?	Characteristics of the included studies?	Scientific quality of the included studies?	Formulated conclusion based on scientific quality of studies?	Appropriate method used to combine findings of studies??	Likelihood of publication bias?	Conflict of interest included?
Huang et al. [29]	YES	YES	YES	U/C	YES	YES	YES	YES	YES	U/C	YES
Zhong et al. [30]	YES	YES	YES	U/C	YES	YES	YES	YES	YES	YES	U/C
Randomized Control Trials (RCTs)											
	Random sequence generation	Allocation concealment	Performance bias	Detection bias	Attrition bias	Reporting bias	Other bias				
Xiao et al. [31]	U/C	LOW	LOW	U/C	LOW	LOW	U/C				
Liu et al. [32]	LOW	LOW	LOW	LOW	LOW	LOW	U/C				
Morelli et al. [33]	LOW	LOW	LOW	U/C	LOW	HIGH	U/C				

Result

**Figure 1:**
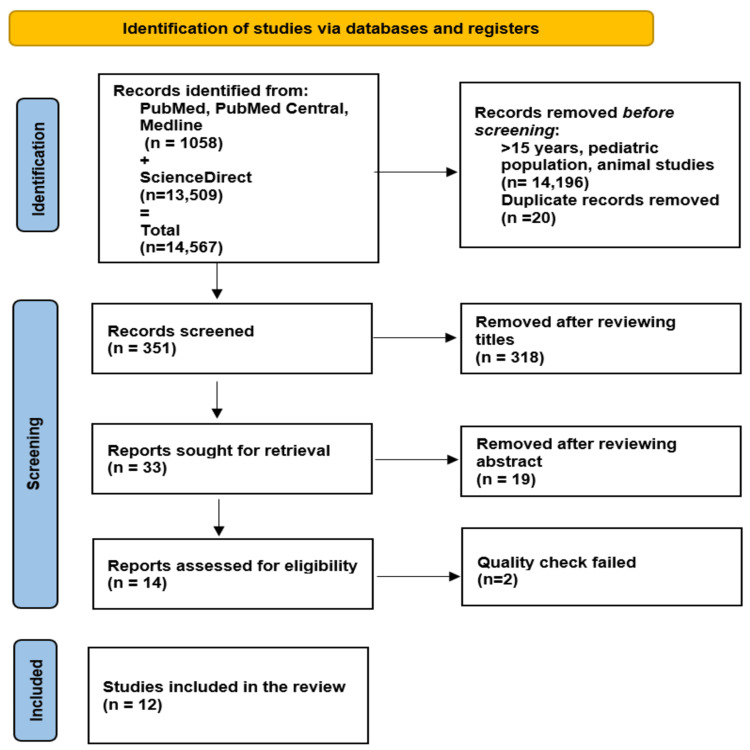
PRISMA flow diagram PRISMA - Preferred Reporting Items for Systematic Reviews and Meta-Analyses

We have gone through 1,058 articles of PubMed, PubMed Central (PMC), Medline, and 13,509 articles of ScienceDirect (total = 14,567). We removed papers that are on the pediatric population, animal studies, and more than 15 years old (total = 14,196). During extraction, we removed all duplicate articles with the help of Microsoft excel. In addition to that, we screen 351 articles through titles and abstracts and excluded 337 articles. Finally, we did a quality check of 14 articles. Of which two failed the quality check, therefore, our final articles are 12 which we reviewed thoroughly. We included articles with almost all study designs. The PRISMA flowchart is in Figure 1.

The summary of included studies is described in Table 3.

**Table 3:** Summary of included studies RCTs - Randomized Control Trials, VP - Vasopressin, TP - Terlipressin, NE - Norepinephrine, MAP - Mean Arterial Pressure, IMCU - Inter-Mediate Care Unit

S.No.	Author	Year	Study Design	Study	Conclusion
1	Nascente et al. [22]	2017	Case-Control study	Used VP as an adjuvant to NE	When the baseline noradrenaline dose is greater than 0.38 mcg/kg/min in patients who have been in septic shock for no more than 48 hours, vasopressin administration is likely to enhance microcirculation.
2	Fawzy et al. [23]	2015	Cohort study	Compared NE and Dopamine as a first line agent	The use of dopamine as the first vasopressor was linked to higher mortality across a number of clinical subgroups.
3	Vail et al. [24]	2016	Cohort study	Epidemiology of VP	Vasopressin was administered to about one-fifth of septic shock patients, but rarely as a single vasopressor.
4	Yerke et al. [25]	2020	Cross-sectional study	Role of plasma concentration of VP	The use of plasma vasopressin concentrations as a therapeutic target to forecast the hemodynamic response to exogenous vasopressin in septic shock is not supported by the findings of this investigation.
5	Ruffin et al. [26]	2018	Case report	Adverse effects of VP	When utilizing vasopressors to treat septic shock, early detection and prompt management of peripheral ischemia are crucial.
6	Kny et al. [27]	2018	Case series	VP in refractory septic shock	When individuals are resistant to norepinephrine, using Vasopressin has little to no effect on mortality.
7	Hallengren et al. [28]	2017	Case series	NE in IMCU	Norepinephrine treatment for elderly septic shock patients showed better than anticipated outcomes in the ward and at 30 day
8	Haung et al. [29]	2020	Systemic Review	Role of TP	The use of Terlipressin was linked to a lower fatality rate in septic shock patients under the age of 60. Terlipressin may also increase peripheral ischemia and improve renal function.
9	Zhong et al. [30]	2020	Meta-Analysis	Role of non-catecholamine vasopressors in combination with NE	Norepinephrine supplementation with non-catecholamine vasopressors was linked to a barely significant decrease in 28-day mortality.
10	Xiao et al. [31]	2016	RCTs	Low dose of TP with NE	Low dose of TP in continuous infusion can help NE achieve the good resuscitation goal by improving tissue blood flow. As the first-line vasopressor for refractory hypotension following severe sepsis or septic shock, low doses of TP may be advised.
11	Liu et al. [32]	2018	RCTs	TP vs. NE	In patients with septic shock, the study found no mortality difference between Terlipressin and Norepinephrine infusion.
12	Morelli et al. [33]	2008	RCTs	TP vs. Dobutamine	Terlipressin (with or without concurrent dobutamine infusion) elevates MAP and significantly lowers norepinephrine needs in human catecholamine-dependent septic shock.

Discussion

Septic shock is the major cause of death in hospital settings mainly in the ICU. 

Management of Septic Shock

**Figure 2:**
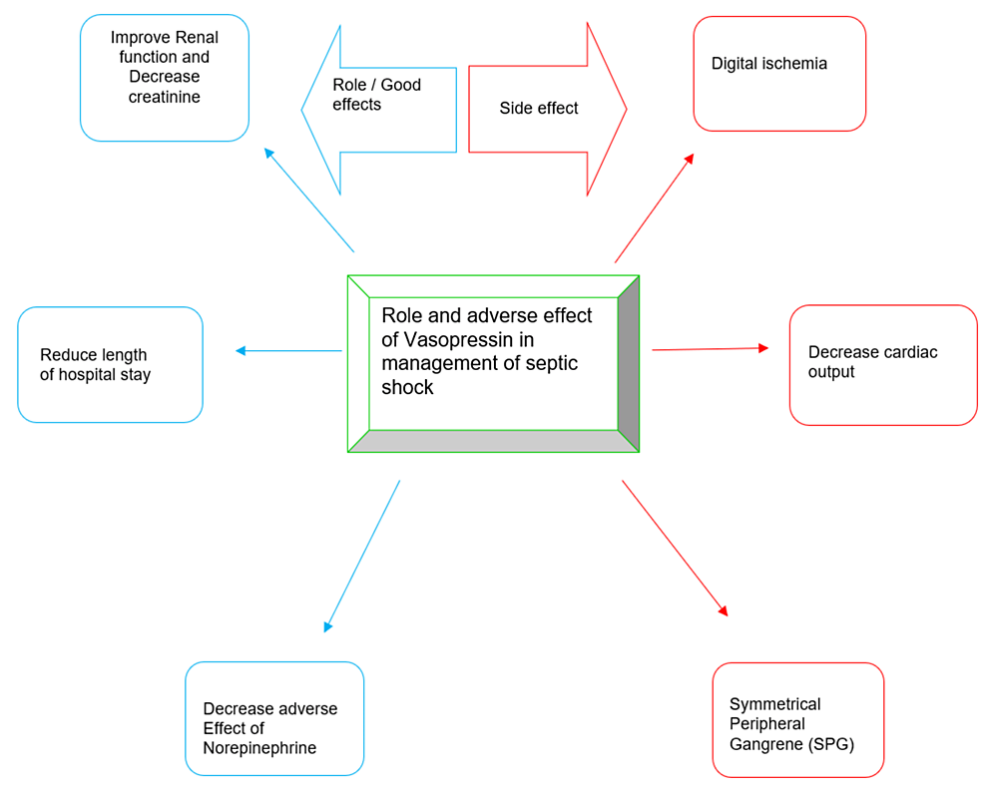
Role and Adverse effect of Vasopressin (VP) in septic shock SPG- Symmetrical Peripheral Gangrene Role and adverse effects of VP are described in detail in the text [22-33]. Image credits: Naishal Mandal and Shaili S. Naik

Managing hypotension is imperative in patients with septic shock for survival. Intravenous fluid, antibiotics, and vasopressors are the mainstay of treatment in a patient with septic shock. There are different types of vasopressors available, that can be used in the treatment of septic shock. However, all vasopressors have a unique mechanism of action through which they have been used in a specific type of shock. For instance, Norepinephrine (NE) is the first-line agent used for the management of septic shock, Dopamine and Dobutamine for cardiogenic shock, and Epinephrine for anaphylaxis shock. Here, in our study, we focused mainly on the role/efficacy and side effects of VP use in septic shock patients. This article compares vasopressors such as NE, VP, Dopamine, and Dobutamine, which are commonly been given to patients with septic shock. The role and adverse effects of VP are described in Figure 2.

VP/Terlipressin(TP)/Selepressin (SP) versus NE

According to a study by Kny et al. [27], VP usage may raise blood pressure and decrease the need for catecholamines in septic shock patients who are resistant to intravenous fluids and other treatments; leading to an indirect reduction in the detrimental effects of catecholamines on renal failure and arrhythmias [27]. The reason not to use NE in higher doses might be the insensitivity of adrenergic receptors in patients with septic shock which diverts NE to work on nonspecific loci leading to adverse effects such as undesirable hemodynamic effects, also enhanced coagulation, reduced innate and adaptive immunity, and increased bacterial replication and virulence [22]. Adrenergic agents increase oxidative stress and have harmful biological effects on the inflammatory response and cell energy metabolisms while non-adrenergic agents might reduce 28-day mortality in patients with septic shock [30]. Nascente et al. [22] studied the effect of short-term infusion of VP in septic shock in patients already taking NE. In this study, they found SP, V1A agonist (a novel selective VP), compared to Terlipressin (TP) improves total vascular density, perfused vascular density, fluid balance, perfusion at microcirculation level and shortens the time of mechanical ventilation (MV) [22,29,32]. The addition of low-dose VP (specifically Arginine VP [AVP]) to NE as an alternative in cases refractory to NE, increases the mean arterial pressure (MAP) and decreases the dose of NE as it potentiates the activity of NE [25]. It has the capacity to improve MAP at the targeted level (>65) which may provide sufficient circulation to splanchnic organs such as the liver (portal vein, hepatic artery) and kidney (by constricting efferent arterioles, increasing glomerular filtration rate (GFR), and decreasing serum creatinine) [33]. When compared to NE, non-catecholamine vasopressors are linked with decreased heart rate (HR) and serum creatinine as well [29,32]. Thus, it was concluded that using TP instead of catecholamine in patients with hepato-renal syndrome might be beneficial. In contrast to this, another study concluded that there has been no significant increase in MAP with TP and no significant decrease in cardiac index (CI) [29]. According to Yerke et al. [25], patients with septic shock initially have higher Endogenous VP concentrations, but over time, the amount of VP in their bodies gradually diminishes, causing their plasma VP concentration to fall. Study shows that plasma VP concentration is not a reliable indicator for use and the hemodynamic response of exogenous VP.

TP in a small dose in continuous infusion in addition to NE can improve survival by increasing tissue perfusion and oxygen delivery, reducing blood lactate level, providing hemodynamic stability, decreasing the occurrence of complications such as cardiac arrest, hypotension, and acute respiratory failure and limiting hospital stay. TP in small doses activates rho-kinase and improves vascular reactivity of septic shock, which increases vascular tone and blood flow. The limitation of this study is a small number of patients were taken, and only renal function tests (RFT), and serum electrolytes [sodium and potassium] were observed. In another study, Zhong et al. [30] experienced a significant reduction in the length of MV, and a nonsignificant decrease in the ICU Length of Stay (ICULOS), Hospital Length of Stay (HLOS), and duration of Continuous Renal Replacement Therapy (CRRT) [30].

Another study done by Kny et al. [27] evaluated the short-term benefit of VP in combination with NE in refractory cases, in view of death rate and length of stay in the ICU. Data on evolution were kept until hospital result (discharge or death) or for up to 30 days following the initiation of VP therapy. In 512 septic patients who utilized VP, 40.6% mortality was confirmed. The current series investigated the use of VP in a condition where NE was ineffective, whereas that meta-analysis included studies in which VP was used as first-line therapy. Within 72 hours of the start of the VP infusion, the majority of the patients passed away. The mortality rate at 30 days was 86.2%. The various vasopressors had similar hemodynamic effects, with NE having some advantages in central venous pressure, urine output, and lactate levels [27]. 

Liu et al. [32] concluded that in general, the use of TP in combination with NE has found no such mortality benefit whereas a study by Kny et al. [27] says that the use of VP in combination with NE improves mortality benefit, and if you use VP in NE refractory cases then the result definitely shows more mortality with VP as patients might already be in deteriorating condition and it's too late to start VP. So, in septic patients with refractory shock treated with VP, early death was higher [27]. Another study by Huang et al. [29] suggested that in patients < 60 years old, the mortality rate was slightly lower in the TP group than in the catecholamine group. Although VP has advantages, the negative impact of increased risk of hyponatremia (not with SP and TP) and digital ischemia with the use of non-catecholamine was also noted [29,30]. In the TP group compared to the NE group, skin ischemia of the limbs, torso, scrotum and abdominal skin is uncommon, and the likelihood of digital ischemia is high. There may be two reasons for digital ischemia with TP one is that combined use with NE in most patients (causes cumulative effect) lead to huge peripheral vasoconstriction and triggers the risk of ischemia. Secondly, using TP in higher doses causes peripheral ischemia [32]. Ruffin et al. [26] identified NE as a contributing factor in developing Symmetrical Peripheral Gangrene (SPG). SPG is a clinical manifestation of an acute onset of ischemia that does not cause any obstruction to the supplying arteries. While fingers and toes are most commonly affected, the least affected ones are the nose, earlobes, and scrotum. The main aim of the study was to draw extra attention to this rare though life-threatening side-effect owing to the fact that it has the potential risk of limb amputation which eventually leads to an impact on the quality of life. They found elevated lactate levels in the patients after initiating. They also revealed that patients developed SPG after receiving low to moderate doses of dopamine, ranging from 2 to 20 μg/kg/min for two days, NE doses ranging from 1 to 30 μg/min for seven days, and VP 2.4 units/hour for three days. They suggested that it is crucial to monitor and treat peripheral ischemia as early as possible while using vasopressors in the treatment of septic shock. While in contrast, Hallengren et al. [28] reported no complication of skin necrosis in the patients treated with NE. They included participants treated with NE in the Inter-Mediate Care Unit (IMCU) for septic shock. Patients were excluded who were treated in the ICU during the same hospitalization. In 91 mostly elderly hospitalized septic shock patients with multiple comorbidities, they found an IMCU mortality of only 27.5%.

Briefly stated, the objective of VP is to augment NE activity, lessen the negative effects brought on by NE alone and in large dosages, improve renal function and lower blood creatinine (thus, it can be utilized in hepatorenal syndrome), and increase survival if given early in adjuvant to NE. VP cannot be given alone as a first-line agent, in the later stage of septic shock, and NE refractory cases since it increases mortality. The drawback of VP is that, in high doses or when combined with NE, it might result in SPG.

NE Versus Dopamine as the First Vasopressor

Fawzy et al. [23] compared NE with Dopamine as an initial vasopressor. Dopamine is commonly used as an alternative to NE in specific situations such as in patients with low risk of arrhythmias, old age, heart failure, valvular disease, coronary artery disease, and patients without a history of atrial fibrillation, venous thromboembolism, or cancer to improve outcome. However, Dopamine can be used as an initial vasopressor in septic shock which also has less acute organ failure, decreases the need for MV at admission, and lower the rate of sepsis resulting from a respiratory or gastrointestinal infection. On the other side, Dopamine may cause new-onset arrhythmias compared to NE. This study failed to find any appropriate reason to prove Dopamine is superior to NE by all the above means. furthermore, the study concluded that Dopamine increases mortality compared to NE in septic shock and Dopamine cannot be used even in patients with a low risk of arrhythmias [23,24]. The limitation of this study is that they could not directly measure the correlation between vasopressor choice. The patient developed SPG after receiving low to moderate doses of Dopamine, ranging from 2 to 20 μg/kg/min for 2 days, and VP 2.4 units/hour for three days [26].

VP versus Dopamine as the Second Vasopressor

A study by Vail et al. [24] compared the use of VP versus Dopamine as a second vasopressor in addition to NE and they found higher rates of arrhythmia and death among patients treated with Dopamine. VP may improve kidney function and reduce tachyarrhythmia at low to moderate infusion rates. VP use was clearly more common among patients with a higher burden of acute organ dysfunction and among those receiving more intensive healthcare including ICU admission, MV, renal replacement therapy, and inotrope treatment. Increased use of inotropes among patients receiving VP causes unopposed vasoconstriction an increase in afterload and a decrease in cardiac output.

Terlipressin (TP) Versus Dobutamine

Morelli et al. [33] observed Dobutamine with high doses causes an increase in heart rate, a decrease in diastolic time led to an increase in oxygen demand of the myocardium, and a decrease in MAP in presence of volume overload. Though Dobutamine has no adverse effects even with higher doses, it is not a good choice for a septic shock as B-adrenergic receptor downregulation impairs signal transduction in sepsis patients, and thus does not increase heart rate. However, has high efficacy in patients with non-septic shock. Although a high bolus dose of TP causes depression in CI, which may be due to baroreceptor activation and an increase in left ventricular afterload, it may be prevented with the continuous low infusion. As TP only targets the V1 receptor, do not cause anti-diuresis. The major findings of this study are: (i) TP markedly increased MAP irrespective of Dobutamine infusion and reduced NE requirement (ii) TP-associated decrease in Venous Oxygen Saturation (SvO_2_) was reversed by high Dobutamine administration.

Limitation

There are different opinions and study results from various studies have been reviewed thoroughly. Firstly, due to the language barrier, we have only included studies from the last 15 years that have been published in English. We have not included the role of vasopressors in pediatric populations. Most of our studies had a small sample size which might cause publication bias. Various studies had different medication dosages, time of administration, and unique combinations. Therefore, the exact prescription of drugs could not be established in our study. Lastly, VP plasma concentration could not be measured might lead to improper measurement of drug effectiveness.

## Conclusions

Following a quality check, all publications included in our study were solely evaluated. Since VP increases MAP and protects renal function, it is effective when given in modest, continuous doses in combination with NE and cannot be recognized as a primary agent for the management of septic shock, according to our analysis of almost all articles. A large dose of either VP or NE, or both, on the other hand, results in severe vasoconstriction and digital ischemia. We may infer that VP is a much superior adjuvant agent than Dopamine and Dobutamine after evaluating all the included articles. The authors are therefore hopeful that this comprehensive assessment will serve as a paradigm for future research that actively explores the knowledge gaps we now have in the management of septic shock and develops guidelines for medical care.
